# The Impact of Quarantine and Physical Distancing Following COVID-19 on Mental Health: Study Protocol of a Multicentric Italian Population Trial

**DOI:** 10.3389/fpsyt.2020.00533

**Published:** 2020-06-05

**Authors:** Vincenzo Giallonardo, Gaia Sampogna, Valeria Del Vecchio, Mario Luciano, Umberto Albert, Claudia Carmassi, Giuseppe Carrà, Francesca Cirulli, Bernardo Dell’Osso, Maria Giulia Nanni, Maurizio Pompili, Gabriele Sani, Alfonso Tortorella, Umberto Volpe, Andrea Fiorillo

**Affiliations:** ^1^Department of Psychiatry, University of Campania “L. Vanvitelli”, Naples, Italy; ^2^Department of Medicine, Surgery and Health Sciences, University of Trieste, Trieste, Italy; ^3^Department of Mental Health, Azienda Sanitaria Universitaria Giuliano Isontina - ASUGI, Trieste, Italy; ^4^Department of Clinical and Experimental Medicine, University of Pisa, Pisa, Italy; ^5^Department of Medicine and Surgery, University of Milano-Bicocca, Milan, Italy; ^6^Center for Behavioral Sciences and Mental Health, National Institute of Health, Rome, Italy; ^7^Department of Biomedical and Clinical Sciences Luigi Sacco, Aldo Ravelli Center for Neurotechnology and Brain Therapeutic, University of Milan, Milan, Italy; ^8^Department of Biomedical and Specialty Surgical Sciences, Institute of Psychiatry, University of Ferrara, Ferrara, Italy; ^9^Department of Neurosciences, Mental Health and Sensory Organs, Faculty of Medicine and Psychology, Sapienza University of Rome, Rome, Italy; ^10^Section of Psychiatry, Department of Neuroscience, University Cattolica del Sacro Cuore, Rome, Italy; ^11^Department of Psychiatry, Fondazione Policlinico Agostino Gemelli IRCCS, Rome, Italy; ^12^Department of Psychiatry, University of Perugia, Perugia, Italy; ^13^Clinical Psychiatry Unit, Department of Clinical Neurosciences, Università Politecnica delle Marche, Ancona, Italy

**Keywords:** pandemic, global mental health, post-traumatic stress disorder, burn-out, anxiety, depression, resilience

## Abstract

The COVID-19 pandemic and its related containment measures—mainly physical distancing and isolation—are having detrimental consequences on the mental health of the general population worldwide. In particular, frustration, loneliness, and worries about the future are common reactions and represent well-known risk factors for several mental disorders, including anxiety, affective, and post-traumatic stress disorders. The vast majority of available studies have been conducted in China, where the pandemic started. Italy has been severely hit by the pandemic, and the socio-cultural context is completely different from Eastern countries. Therefore, there is the need for methodologically rigorous studies aiming to evaluate the impact of COVID-19 and quarantine measures on the mental health of the Italian population. In fact, our results will help us to develop appropriate interventions for managing the psychosocial consequences of pandemic. The “COVID-IT-mental health trial” is a no-profit, not-funded, national, multicentric, cross-sectional population-based trial which has the following aims: a) to evaluate the impact of COVID-19 pandemic and its containment measures on mental health of the Italian population; b) to identify the main areas to be targeted by supportive long-term interventions for the different categories of people exposed to the pandemic. Data will be collected through a web-platform using validated assessment tools. Participants will be subdivided into four groups: a) Group 1—COVID-19 quarantine group. This group includes the general population which are quarantined but not isolated, i.e., those not directly exposed to contagion nor in contact with COVID-19+ individuals; b) Group 2—COVID-19+ group, which includes isolated people directly/indirectly exposed to the virus; c) Group 3—COVID-19 healthcare staff group, which includes first- and second-line healthcare professionals; d) Group 4—COVID-19 mental health, which includes users of mental health services and all those who had already been diagnosed with a mental disorder. Mental health services worldwide are not prepared yet to manage the short- and long-term consequences of the pandemic. It is necessary to have a clear picture of the impact that this new stressor will have on mental health and well-being in order to develop and disseminate appropriate interventions for the general population and for the other at-risk groups.

## Background

The ongoing COVID-19 pandemic represents an unprecedented event in terms of consequences for physical and mental health of individuals and for the society at large (1–4). In order to reduce the spread of the virus, national and international bodies and institutions have ordered quarantine, physical distancing, and isolation almost everywhere in the world. However, the psychological consequences of quarantine, such as frustration, loneliness, and worries about the future are well-known risk factors for several mental disorders, including anxiety, affective disorders, and psychoses (5–7).

From a medical and sociological viewpoint, the pandemic caused by COVID-19 represents a unique event, since it does not resemble any other previous traumatic event, such as earthquakes or tsunamis (8). In those cases, the traumatic factors are usually limited to a specific area and to a given time; affected people know that they can “escape” from the event. On the contrary, in the case of COVID-19 pandemic, the “threat” can be everywhere and can be carried by every person next to us (9–11). Therefore, people living in cities most severely impacted by the pandemic are experiencing extremely high levels of uncertainties, worries about the future and fear of being infected.

The only comparable studies are those carried out during the SARS outbreak (12–16). Those studies showed that people experienced fear of falling sick or dying, feelings of helplessness, increased levels of self-blame, fear, and depression (17–20). During quarantine and physical distancing, Internet and the social media can be useful in reducing isolation and increasing opportunities to keep in contact with family members, friends, and co-workers at any time (21, 22). However, Internet may also represent a risk factor for mental disorders, in particular Internet Gaming Disorder. Moreover, Internet can also have a negative impact on mental health of the most vulnerable people, such as those who live alone or the elderly, since it spreads an uncontrolled amount of information (a situation known as “infodemic”).

In the current pandemic, the impact of quarantine and physical distancing on the mental health of the general population has been explored only in a few studies, mostly conducted in China, where the pandemic started (23–25). Qiu et al. (26) found that 35% of the population experienced psychological distress; in particular, those more vulnerable to stress and more likely to develop post-traumatic stress disorder were women and individuals aged between 18 and 30 years or older than 60 years. Moreover, people were more concerned about their own health and that of their family members, while less concerned about leisure activities and relationships with friends (24, 27).

After China, Italy has been the first country to face the contagion of COVID-19 and one of the countries with the highest number of deaths due to this coronavirus (http://www.salute.gov.it/portale/nuovocoronavirus/). On March 8, the lockdown status has been declared by the Italian government. This status included the definition of specific containment and quarantine measures, such as the interdiction of all public meetings and strict movement restrictions (i.e., possibility to go out only for working, serious health reasons, or other urgent needs). These containment measures have been prolonged until May 4.

Moreover, the expected psychosocial and emotional reactions to the pandemic observed in the general population may be significantly different in the Chinese and Italian populations due to their socio-cultural characteristics and historical contexts, which obviously impact on people’s behaviors and attitudes. Furthermore, the organization of public health system is different in Italy compared to China and other Eastern Asian countries, also due to financial constraints. In fact, although in those countries the model of care has shifted in the last 20 years to become more similar to a Western model of care, it has to be acknowledged that 20 years is a relatively short period of time, and differences may still persist.

Methodologically rigorous studies are needed in order to evaluate the impact of COVID-19 and quarantine measures on the mental health of Italian population. These data will help us to develop appropriate interventions for managing the psychosocial consequences of the pandemic (28–30). The present study has been developed with the aims to: a) evaluate the impact of COVID-19 pandemic and its containment measures on mental health of the Italian population; b) to identify the main areas to be targeted by supportive long-term interventions for the different categories of people exposed to the pandemic.

## Methods

### Design

The “COVID-IT-mental health trial” is a no-profit, not-funded, national, multicentric, cross-sectional population-based trial involving the following eleven sites: University of Campania “Luigi Vanvitelli” (Naples), Università Politecnica delle Marche (Ancona), Università Milano Bicocca, Università “Statale” (Milan), University of Perugia, University of Pisa, Sapienza University of Rome, “Cattolica” University of Rome, University of Trieste, University of Ferrara; the Center for Behavioral Sciences and Mental Health of the Istituto Superiore di Sanità (Rome). The Department of Psychiatry of the University of Campania “Luigi Vanvitelli” in Naples is the coordinating center, which has originally conceived the study idea and design.

### Data Collection

#### Recruitment Procedure

An online survey has been set up through EUSurvey, a web platform launched in 2013 by the European Commission. The application, hosted at the Department for digital services (DG DIGIT) of the European Commission, is available to all EU citizens at https://ec.europa.eu/eusurvey. The survey will be online from March 30 to June 30, 2020 (https://ec.europa.eu/eusurvey/runner/COVIDSurvey2020). The survey takes approximately 15–30 min to be completed. Participants can stop the survey at any time and save their answers as “draft” on the web-platform. Furthermore, participants can interact with the principal investigator of the study and with all researchers through email messages at any time during and after study participation.

Participants will be subdivided into four groups: a) Group 1—COVID-19 quarantine group. This group includes the general population which are quarantined but not isolated, i.e., those not directly exposed to contagion nor in contact with COVID-19+ individuals; b) Group 2—COVID-19+ group, which includes isolated people directly/indirectly exposed to the virus; c) Group 3—COVID-19 healthcare staff group, which includes first- and second-line healthcare professionals; d) Group 4—COVID-19 mental health, which includes users of mental health services and all those who had already been diagnosed with a mental disorder.

The survey addresses the Italian population aged over 18 years through a multistep procedure: 1) email invitation to health professionals and their patients; 2) dissemination of the link through social media channels (Facebook, Twitter, Instagram) and the mailing lists of national psychiatric associations; 3) involvement of national associations of stakeholders (e.g., associations of users/carers); 4) official communication channels (e.g., university websites; websites of the hospitals directly involved in the management of the pandemic).

The invitation letter includes information on study purposes and confidentiality. The provision of the informed consent is mandatory in order to start the survey.

The snowball sampling procedure—without the definition of strict inclusion/exclusion criteria (except that of age limit)—will give us the opportunity to recruit a large sample of the Italian population and to evaluate the effect of the studied variables on the outcome measures.

#### Assessment Instruments

The survey includes the following self-reported questionnaires: the General Health Questionnaire - 12 items (GHQ-12) (31); the Depression, Anxiety and Stress Scale - 21 Items (DASS-21) (32); the Obsessive-Compulsive Inventory – Revised (OCI-R) (33); the Insomnia Severity Index (34); the Severity-of-Acute-Stress-Symptoms-Adult (35); the Suicidal Ideation Attributes Scale (SIDAS) (36); the Impact of Event Scale - 6 items (37); the UCLA loneliness scale - short version (38); the Brief COPE (39); the Post Traumatic Growth Inventory short form (40); the Connor-Davidson Resilience Scale – short form (41); the Multidimensional Scale of Perceived social support (42); the Pattern of Care Schedule (PCS)—modified version (43); the Maslach Burnout Inventory (only for health professionals) (44). Respondents’ main socio-demographic characteristics, as well as data on their Internet use, will be collected through an *ad hoc* schedule. All assessment instruments used for the study are detailed in **Table 1**.

**Table 1:** Assessment tools used in the survey.

Assessment tool	Acronym	N. items	Description
General Health Questionnaire-12	GHQ-12	12	Each item assesses the severity of a mental problem on a 4-level Likert scale. The total score ranges from 0 to 36, with higher scores indicating worse conditions.
Depression, Anxiety and Stress Scale - 21	DASS-21	21	It consists of three subscales. The depression subscale assesses dysphoria, hopelessness, devaluation of life, self-deprecation, lack of interest/involvement, anhedonia, and inertia. The anxiety scale assesses autonomic arousal, skeletal muscle effects, situational anxiety, and subjective experience of anxious affect. The stress scale assesses difficulty in relaxing, nervous arousal, and being easily upset/agitated, irritable/over-reactive and impatient.
Obsessive-Compulsive Inventory – Revised	OCI-R	18	Each item assesses the severity of obsession or compulsion on a 5-level Likert scale. The total score range from 0 to 72, with higher scores indicating worse conditions.
Insomnia Severity Index	ISI	7	Each item assesses the nature, severity, and impact of insomnia on a 5-level Likert scale. The aspects evaluated includes sleep onset, sleep maintenance, and early morning awakening problems, sleep dissatisfaction, interference of sleep difficulties with daytime functioning, noticeability of sleep problems by others, and distress caused by the sleep difficulties. The total score ranges from 0 to 28.
Severity-of-Acute-Stress-Symptoms-Adult	SASS	9	It assesses the severity of post-traumatic stress disorder in adult individuals. Each item assesses the severity of post-traumatic symptoms during the past seven days.
Suicidal Ideation Attributes Scale	SIDAS	5	It assesses all the attributes of suicidal thoughts: frequency, controllability, closeness to attempt, level of distress associated with the thoughts, and impact on daily functioning. Each item is assessed on 10-level Likert scale. When the score at the first item is zero, the remaining items are not compiled.
Impact of Event Scale-6	IES-6	6	It assesses the impact of the traumatic event, including three subscales that describe the three major symptoms of posttraumatic stress: intrusion, avoidance, and hyperarousal.
UCLA loneliness scale - short version	UCLA	8	It is an 8-item scale designed to measure one’s subjective feelings of loneliness as well as feelings of social isolation.
Brief Coping Orientation to Problems Experienced	Brief-COPE	28	It includes 14 subscales designed for measuring effective and ineffective ways to cope with a stressful life event. The subscales include: self-distraction, active coping, denial, substance use, use of emotional support, use of instrumental support, behavioral disengagement, venting, positive reframing, planning, humor, acceptance, religion, and self-blame.
Post Traumatic Growth Inventory- short form	PTGI	10	It evaluates the construct of post-traumatic growth on a 6-level Likert scale.
Connor-Davidson Resilience Scale – short form	CD-RISC	10	It evaluates the levels of resilience and it includes the following five factors: personal competence, high standards, and tenacity; trust in one’s instincts, tolerance of negative affect, and strengthening effects of stress; positive acceptance of change and secure relationships; control; spiritual influences. Each item is rated on a 6-level Likert scale.
Multidimensional Scale of Perceived Social Support	MSPSS	12	It evaluates the levels of perceived adequacy of social support from the family, friends, and significant others on a 5-level Likert scale
Pattern of Care Schedule - modified version	PCS	20	It is an *ad hoc* schedule evaluating the pharmacological and nonpharmacological treatments received by participants
Maslach Burnout Inventory (only for healthcare professionals)	MBI	22	It evaluates the three dimensions of burnout: emotional exhaustion, depersonalization, and personal accomplishment

### Outcomes

#### Primary Outcome

The primary outcome of the study is the global score at the DASS-21. This choice is due to the fact that this assessment measure has already been used in a large population study carried out in China, thus giving us the opportunity to compare the Italian situation with the Chinese one (45). Our study hypothesis is that the pandemic and the related containment measures are associated with higher levels of depressive and anxiety symptoms in the surveyed population compared to a community Italian sample not exposed to the pandemic (46). Furthermore, a significant difference between groups will be identified (COVID-19 quarantine group = COVID-19 healthcare professional second-line < COVID-19+ group = COVID-19 healthcare professional first-line group < COVID-19 mental health group).

#### Secondary Outcomes

In the COVID-19 quarantined group, the severity of obsessive-compulsive symptoms, evaluated through the OCI-R, the perceived loneliness and suicidal ideation will be considered as secondary outcome measures.

In the COVID-19+ patient group, the severity of post-traumatic symptoms at the Severity-of-Acute-Stress-Symptoms-Adult scale will be considered. The hypothesis is that post-traumatic symptoms are more severe in this group compared to the other ones.

In the COVID-19 health staff group, the presence of burn-out symptoms, in particular mental exhaustion, and suicidal ideation will be considered. We anticipate that first-line professionals will report higher levels of mental exhaustion and suicidal ideation compared to second-lines staff members.

In the COVID-19 mental health group, the secondary outcome measures will include the adoption of maladaptive coping strategies (e.g., drinking alcohol) and a poor resilience style. Patients with pre-existing mental disorders are expected to adopt more maladaptive coping strategies and poorer resilience styles compared to the other three groups.

#### Exploratory Outcomes

**Figure 1:**
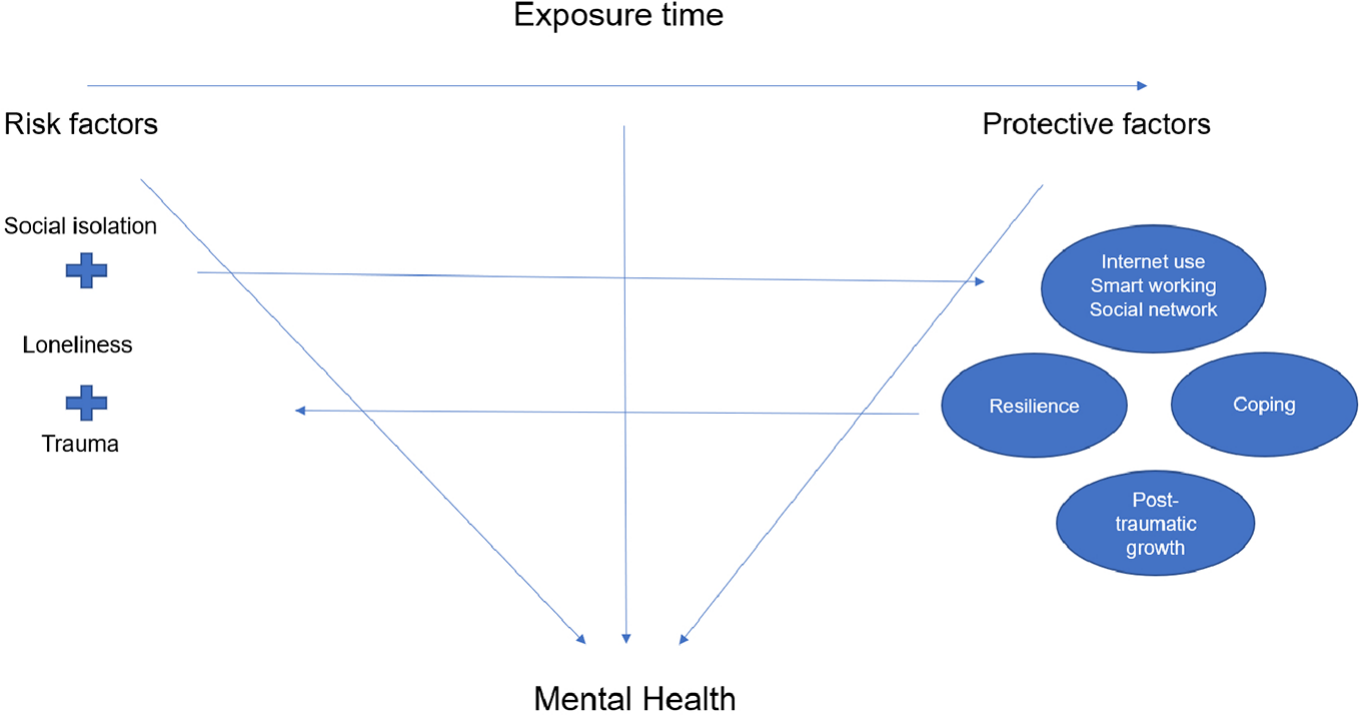
Determinants of mental health during the COVID-19 pandemic.

The use of Internet and social media will be tested as possible moderator of the impact of pandemic and quarantine (**Figure 1**). Moreover, the exposure time to COVID-19 and to the related containment measures will be tested as possible mediators of the severity of the clinical symptomatology. Finally, the other exploratory outcomes will include the variety of coping strategies and resilience styles as well as the different levels of post-traumatic growth.

### Data Analysis

Statistical analyses will be conducted according to a multistep plan. Missing data will be handled using the multiple imputation approach (47). Descriptive statistics will be calculated for the dependent and confounding variables. A bilateral alpha of 0.05 is considered, and error and confidence intervals are calculated at 95%.

The analytic plan will include: 1) data cleaning of the online dataset and replacement of missing values; 2) descriptive statistics of the general characteristics of the recruited sample, in terms of levels of depressive and anxiety symptoms, post-traumatic and stress-related symptoms, insomnia, satisfaction with life, suicidal ideation, hopelessness, post-traumatic growth, resilience, coping strategies, and social support; 3) sub-groups analyses based on the level of exposure to the pandemic (i.e., COVID-19 quarantine group vs. COVID-19+ patients group vs. COVID-19 healthcare staff group vs. COVID-19 mental health group); 4) calculation of a propensity score, in order to adjust our findings for the likelihood of being exposed to the pandemic and to the quarantine (48, 49). This method is adopted since it produces a better adjustment for differences at baseline, rather than simply including potential confounders in the multivariable models. The independent variables used for calculating the propensity score will include gender, age, socio-economic status, and geographical region. The obtained propensity score will be used to weight the observations in the multivariable analyses. In the final regression model, the inverse probability weights, based on the propensity score, will be applied in order to model for the independence between exposure to the pandemic/quarantine and mental health outcomes and estimation of causal effects (48, 49); 5) development of a Structural Equation Model (SEM), in order to evaluate the possible role as mediators and moderators of coping strategies, post-traumatic growth and usage of social networks on the severity of depressive and anxiety symptoms, post-traumatic and stress-related symptoms, suicidal ideation, and hopelessness.

In order to improve the external validity and generalizability of our findings, all analyses will be controlled for the impact of confounding variables, such as age, gender, and geographical region.

Data will be stored in an online dataset by the coordinating center. For safety reasons, the dataset will be protected by a two-step password. It will be possible to export data in compatible formats with common calculation software (e.g., Microsoft Access and Excel) and in specific softwares (e.g., SPSS and STATA) for the statistical analyses.

### Ethics and Dissemination

This study is being conducted in accordance with globally accepted standards of good practice, in agreement with the Declaration of Helsinki and with local regulations. The study protocol has been approved by the Ethical Review Board of the University of Campania “L. Vanvitelli” (Protocol number: 0007593/i).

## Discussion

Our survey will give us the opportunity to describe the impact of the pandemic on the mental health of different subgroups of the Italian population.

In fact, the analyses will be run according to the four subgroups of respondents: the general population not directly affected by the virus (COVID-19 quarantine group); people who have had a direct or indirect contact with the virus (COVID-19+ patients group); those working in health care units as first or second-line staff (COVID-19 healthcare staff group); people with mental health problems, independently from the contact with the virus (COVID-19 mental health). This choice is due to the evidence that stress and traumas have a different impact on different target groups (7, 50–52).

In the COVID-19-quarantine group, we anticipate that the pandemic and the related containment measures will increase the levels of stress, anxiety and depression, as well as other stress-related symptoms. In particular, physical distancing has obviously changed the patterns of daily routine in order to mitigate the spread of the disease, with serious consequences on mental health and well-being in both the short- and long-term (53). Similar consequences would require immediate efforts for developing preventive strategies as well as direct interventions aiming to mitigate the impact of the outbreak on individual and population mental health. The longer the pandemic will last the most the ordinary life of the general population will be seriously affected. In particular, Zhang et al. (23) have highlighted the need to pay attention to the mental health of people who have not been directly infected by the virus though have been forced to stop all their activities during the outbreak. These people represent the most susceptible group to the detrimental impact of quarantine and physical distancing measures adopted during the lockdown. Moreover, during the current pandemic, it is reasonable to expect that the incidence of severe mental disorders will increase, but also that of other mental health disturbances not reaching the threshold for a full-blown diagnosis (3). However, currently available data are based on studies carried out in China and the different socio-cultural context may limit the generalizability of findings to the Italian and Western contexts. Therefore, we consider essential to collect Italian data in order to develop data-driven guidelines for an adequate management of mental health problems during the emergency and the post-emergency phases. In fact, this survey will represent the starting point for developing, validating, and implementing psychosocial supportive interventions (53, 54), as discussed later in this paper.

We hypothesized that Internet and social media can play a buffering role in the development of psychiatric symptoms (25, 55). It may be that online contacts and interactions will limit the detrimental effects of social isolation (56). Moreover, Internet can represent the ideal setting for providing supportive interventions through tele-mental health applications (57–60). However, the positive effect of Internet and social media has to be confirmed yet, since it is only speculative at this stage.

In the COVID-19+ patient group (i.e., those with a direct or indirect contagion), the impact on mental health has been mostly neglected during the acute emergency phase. Of course, this has been due to the fact that the infection is a potentially life-threatening condition, as confirmed by the need for hospitalization in intensive care units for many patients (61). In particular, the experience of being isolated in the hospital, the perceived danger, uncertainty about own physical conditions and the fear of dying alone can be considered risk factors for the development of post-traumatic, anxiety, and depressive symptoms (62, 63). The only study conducted in China so far has documented that over 90% of COVID+ patients admitted to the hospital reported significant post-traumatic stress symptoms (62, 64, 65). Furthermore, the authors found that providing patients with psychoeducational intervention is well received and perceived as helpful and useful by users.

As regards the effects on mental health of those working in health care units as first-line or second-line staff (COVID-19 healthcare staff group), we expect that many health professionals will experience symptoms of burn-out, including mental exhaustion, irritability, detachment from reality, and insomnia. In a survey involving medical and non-medical health workers, Zhang et al. (23) found a higher prevalence of insomnia, anxiety, depressive symptoms, somatization, and obsessive-compulsive symptoms in mental health staff. Moreover, front-line medical staff working in close contact with infected patients (e.g., staff professionals working in the departments of respiratory, emergency, infectious disease, and intensive care unit) showed higher scores on depressive/anxiety symptoms and had a twofold increase in risk to develop a mental health problem (66–69). However, the effect on suicidal ideation of health professionals has not been investigated yet and will be the focus of one of our work-packages.

Finally, the pandemic will affect the mental health status of people who already suffer from mental health problems, independently from the contact with the virus (COVID-19 mental health group). Although the effects of the coronavirus on mental health have not been systematically studied, it is likely that the COVID-19 will have detrimental effects on patients with pre-existing mental health problems. Many patients with severe mental disorders have been overlooked during the pandemic, although they can have a higher risk of contracting the virus and of death considering the higher prevalence of somatic comorbidities compared to general population and the difficulties in accessing health services (70).

However, if protracted, social isolation may increase the risk of recurrences of episodes of mental disorders, beyond triggering the onset of new mental disorders in most vulnerable people. Moreover, objective social isolation and subjective feelings of loneliness are associated with a higher risk of suicidal ideation and suicide attempts (71). For many persons with mental disorders, being alone is a heavy burden, far beyond that experienced by many other persons (72).

In patients with pre-existing anxiety disorders or obsessive-compulsive disorder, we expect an exacerbation or worsening of their clinical symptoms. Moreover, the fact that there is not (yet) a definitive treatment for the COVID infection represents another potential stressor, further increasing the levels of anticipatory anxiety and reducing personal functioning. In our study, both obsessive-compulsive and anxiety symptom clusters will be evaluated through reliable and validated questionnaires.

We believe that our study has several strengths, which should be highlighted. First, this is the first national multicentric, no-profit study carried out in Italy with a rigorous methodology for evaluating the impact of pandemic and quarantine on mental health. Second, the development of a web-based platform for data collection will give us the opportunity to recruit a high number of participants. Based on previous population surveys carried out in Italy, an ideal target would have been 10,000 participants, but this target has been reached in only 7 days. Therefore, we expect to reach more than 20,000 people within the study period. A third relevant strength of our study is the selection of validated and reliable assessment instruments, which are available and validated in several languages. The next step of the project will be to adapt our survey to the European level, by involving several countries. Fourth, several psychopathological dimensions will be evaluated, not only those usually assessed following natural disasters, such as the post-traumatic and depressive-anxious dimensions. In this study, we will also evaluate the obsessive-compulsive spectrum, the suicidal ideation, the maladaptive use of Internet, among the others, which represent novel targets for psychiatrists (73, 74).

Our study has obviously also some limitations. In particular, the study sample includes the adult population only, due to existing restrictions related to the provision of informed consent of children and adolescents in Italy. However, it is likely that the pandemic will have a detrimental impact on the mental health of adolescents as well (75, 76). Moreover, being exposed to a traumatic event during early life is associated with alterations in the social, emotional, and cognitive development and could determine a variety of impairment in the adulthood. The effects of the pandemic on children and adolescents will be evaluated in an *ad hoc* study, in which we will explore the relationship between parents and their underage children during the pandemic. Another limitation is related to the recruitment process, which might partially bias our findings, since only persons interested in the topic of the survey may have voluntarily participated. However, we expect that most people are interested in participating in the survey given the global magnitude of the current traumatic threat with collective psychological and social reactions.

Another possible limitation of our study is the choice to use a web-based online survey, which may have limited the participation of people not having access to the Internet or not familiar with online tools, particularly the elderly. The cross-sectional design of the study does not allow an evaluation of changes over time as regards the levels of severity of symptoms. However, in order to overcome this possible bias, we will compare our findings with those already available from the Italian population (46) and will adopt a propensity score approach in order to understand the impact of the duration of exposure to the pandemic on the risk of developing psychiatric symptoms. With this methodology, we will be able to evaluate the levels of post-traumatic growth and the type of resilience styles in the study population in order to identify possible critical areas to be targeted in the post-acute phase. However, these psychological constructs are slow to change, and this is why we will promote a second wave of the survey, which will start six months after the end of the “lockdown phase” in Italy. Finally, the survey link can be used multiple times in order to allow sharing and re-posting it. This methodological choice could bias the findings, since the same person can potentially compile the survey several times. However, this methodological choice was due to the adoption of the “snowball” sampling, and it is rather unlikely that someone can compile the same long survey more than once.

### Next Steps

Based on the findings of this study and on our previous work in the development of psychosocial interventions (77–79), we aim to develop a psychosocial intervention which will include elements of classic psychoeducation, cognitive-behavioral therapy, and motivational intervention (80–84). In particular, we are developing an experimental intervention which includes information on the mental health consequences of the pandemic and on strategies to prevent them; practical advices for promoting healthy lifestyle behaviors (e.g., healthy eating, regular sleeping patterns, physical activity, etc.); stress-management techniques; communication strategies; problem-solving skills. Based on participants’ needs, additional sessions on suicide prevention, burn-out, and Internet dependence may be provided.

The intervention will include face-to-face sessions and tele-mental health sessions (85, 86). Information will be provided through instant messages (e.g., Chatbot), email contacts, and the development of an *ad hoc* app.

The modules of the intervention will be adapted according to the characteristics and the needs of the four above-mentioned target groups. In particular, in the COVID-19 quarantine group, the main focus of the intervention will be the improvement of healthy lifestyle behaviors; for the COVID-19+ patients group, the intervention will include a specific focus on post-traumatic symptoms and on the risk of being socially stigmatized; for the COVID-19 healthcare staff group, specific sessions will be dedicated to the burn-out syndrome and the management of stressful situations; for the COVID-19 mental health group, sessions on resilience, coping strategies, and the detection of early warning signs of relapses will be included.

The proposed experimental intervention will be tested in a randomized controlled trial which will start when the acute phase of the pandemic will be over, and the control group will be represented by an informative group intervention on the effects of the pandemic on mental health.

Moreover, our survey is going to be translated into different languages in order to assess the impact of the pandemic in other European countries.

## Conclusions

The pandemic and the quarantine may have a detrimental impact on mental health. An increase of psychiatric symptoms and of mental health problems in the general population is expected. Most health professionals working in isolation units and resuscitation departments very often do not receive any training or support for their mental health care. Mental health services worldwide are not prepared to manage the short- and long-term consequences of pandemic. It is necessary to have a clear picture of the impact that these new stressors are having on mental health and well-being in order to develop and disseminate appropriate preventive interventions for the general population as well as for the different at-risk groups.

## Ethics Statement

This study is being conducted in accordance with globally accepted standards of good practice, in agreement with the Declaration of Helsinki and with local regulations. The study protocol has been approved by the Ethical Review Board of the University of Campania “L. Vanvitelli” (Protocol number: 0007593/i).

## Author Contributions

VG, GaiS, ML, VV, and AF designed the study and wrote the protocol. UA, GC, CC, FC, BDO, MN, MP, GabS, AT, and UV revised the draft of the paper. All authors contributed to the article and approved the submitted version.

## Conflict of Interest

The authors declare that the research was conducted in the absence of any commercial or financial relationships that could be construed as a potential conflict of interest.

The handling editor declared a past co-authorship with several of the authors, AF, MP, UV, GS.
